# Man With Bilateral Leg Swelling

**DOI:** 10.5811/cpcem.2019.9.43698

**Published:** 2019-10-14

**Authors:** Jake Toy, Alexander Garrett, Yiju Teresa Liu

**Affiliations:** Harbor-UCLA Medical Center, Department of Emergency Medicine, Torrance, California

## Abstract

A 52-year-old man without known medical history presented with painful, progressive, bilateral lower extremity edema over a two-week period. An abdominal exam noted a firm left upper quadrant mass. Emergency department (ED) point-of-care ultrasound (POCUS) revealed a hyperechoic, heterogeneous structure in the inferior vena cava that was determined to represent a tumor thrombus extending from a primary renal cell carcinoma. This case demonstrates how POCUS was valuable in rapidly diagnosing this rare cause of lower extremity edema and its usefulness in directing the initial ED management of this patient.

## CASE PRESENTATION

**Image 1:**
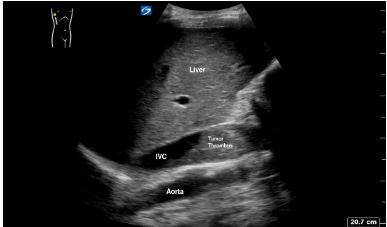
Point-of-care ultrasound of the abdomen demonstrating a hyperechoic, heterogeneous structure in the inferior vena cava.

A 52-year-old man with no medical history presented to the emergency department (ED) with painful, progressive, bilateral lower extremity edema over a two-week period. The patient also had the following complaints: difficulty with ambulation; blood in his urine; dull, left lower quadrant abdominal pain for one month; and an 80-pound weight loss over two years. Vital signs were within normal limits. Palpation of the abdomen revealed a large, firm mass in the left upper quadrant and 2+ pitting edema in the bilateral lower extremities up to the mid-shin. Point-of-care ultrasound (POCUS) of the heart and lower extremities were unremarkable. Abdominal POCUS revealed a hyperechoic, heterogeneous structure in the inferior vena cava (IVC) (Image 1 and Video).

## DISCUSSION

**Image 2:**
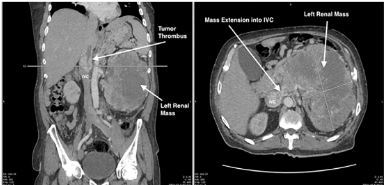
Computed tomography abdomen and pelvis shown in the coronal (left) and axial (right) view demonstrating a large heterogeneous structure measuring approximately 20 × 14 × 19 centimeters and extending into the inferior vena cava.

ED POCUS revealed a hyperechoic, heterogeneous structure in the IVC representing a tumor thrombus, which often results after intravascular extension of a primary tumor. This occurs most commonly in the setting of the following: renal cell carcinoma (RCC); hepatocellular carcinoma; Wilms tumor; and adrenal cortical carcinoma.^1^ Computed tomography demonstrated continuity between a large heterogeneous left kidney mass and tumor thrombus in the IVC (Image 2). The patient was ultimately diagnosed with high-grade clear cell RCC with tumor thrombus extension into the IVC likely resulting in his bilateral leg swelling.

To date, no previous images in the literature have demonstrated POCUS identification of an IVC tumor thrombus in the ED. Radiology literature dating back to the 1980s attempted to characterize known IVC tumor thrombi with ultrasound.^2^ In emergency medicine literature, two case reports describe the detection of IVC thrombosis during an ED POCUS exam, although neither was associated with extension from intra-abdominal malignancy.^3^,^4^ In this case, POCUS allowed clinicians to quickly identify the likely etiology of this patient’s lower extremity swelling, and guided initial clinical management and additional advanced imaging decisions.

**CPC-EM Capsule**

What do we already know about this clinical entity?

*No known reports to date have published images depicting an inferior vena cava (IVC) tumor thrombus detected on emergency department point-of-care ultrasound (POCUS)*.

What is the major impact of the image(s)?

*This case describes how POCUS was used to rapidly identify an IVC tumor thrombus as the etiology of bilateral lower leg swelling, which in turn guided initial management*.

How might this improve emergency medicine practice?

*The case emphasizes that POCUS is an effective and versatile tool to make rapid diagnoses in the emergency department*.

## Supplementary Information

Video: Point-of-care ultrasound of the abdomen.
